# Role of Charlson comorbidity index in predicting intensive care unit readmission in patients with aortic aneurysm

**DOI:** 10.1097/MD.0000000000040033

**Published:** 2024-11-01

**Authors:** Yu-Fei Zhan, Feng Li, Long-Chuan Wu, Lin Chen, Can-Yan Zhu, Ming-Shuai Han, Guo-Fang Ma, Yong-Hong Zhong

**Affiliations:** a Emergency Medicine, Linping Campus, The Second Affiliated Hospital of Zhejiang University School of Medicine, Hangzhou, Zhejiang, China; b Respiratory Medicine, Linping Campus, The Second Affiliated Hospital of Zhejiang University School of Medicine, Hangzhou, Zhejiang, China.

**Keywords:** aortic aneurysm, Charlson comorbidity index, ICU readmission, MIMIC, prediction

## Abstract

The purpose of this study was to investigate the value of the Charlson comorbidity index (CCI) in predicting intensive care unit (ICU) readmission in aortic aneurysm (AA) patients. Patient information came from the Medical Information Mart for Intensive Care- IV (MIMIC-IV) database. The relationship between CCI and ICU readmission was analyzed by restricted cubic spline, generalized linear regression, trend analysis, and hierarchical analysis. The clinical value of CCI in predicting ICU readmission was analyzed by receiver operating characteristic curve, decision curve analysis, XGBoost regression, and random forest regression. A total of 523 patients with AA were enrolled in the study. Patients with AA who were readmitted to the ICU had higher width of red blood cell distribution width (RDW) and higher CCI. CCI had better performance and clinical net benefit for predicting ICU readmission than RDW. An independent nonlinear relationship was found between CCI and ICU readmission. The trend analysis suggested that the risk of ICU readmission increased with higher CCI scores. The hierarchical analysis showed that their association was mainly found in surgery requirement populations regardless of AA types. Further, CCI was found to have better clinical value in predicting ICU readmission of thoracic aortic aneurysm (TAA) patients undergoing surgery. Age, renal disease, chronic lung disease, and dementia were important components of CCI in predicting ICU readmission of TAA patients undergoing surgery. CCI was independently associated with the ICU readmission of AA patients in a positive relationship and had more favorable prediction performance in TAA patients who underwent surgery.

## 1. Introduction

Aortic aneurysm (AA) is a condition where the aorta experiences pathological dilatation, either locally or diffusely, involving the entire aortic wall. The diameter of the aneurysm is 1.5 times or greater than the normal aortic diameter. It commonly includes thoracic AA (TAA) and abdominal aortic aneurysm (AAA) in clinical practice. Common causes include hypertension, atherosclerosis, infection, trauma, systemic rheumatic diseases, and connective tissue diseases. AA has an insidious onset and is usually asymptomatic. However, once the aneurysm ruptures, it can be life-threatening, with a mortality rate of up to 90%. The current treatment for AA is limited to surgical repair. Patients with AA often require intensive care unit (ICU) admission for postsurgical monitoring due to the high mortality rate.^[1]^ The ICU is a vital medical resource for patients. But it is also a costly 1. The cost of the ICU was 4 to 6 times that of the general ward.^[2,3]^ Compared to patients who only stayed in the ICU once, those who required readmission had higher mortality rates, longer hospital stays, and increased medical expenses.^[4–6]^ A study conducted in 35 hospitals across the United States revealed that patients who were readmitted to the ICU had a 4-fold increase in the probability of hospitalization death and a 2.5-fold increase in length of stay, compared to those who only stayed in the ICU once.^[7]^ ICU readmission can lead to additional costs and resource use for hospitals, which can place a financial burden on healthcare organizations.^[8]^ Therefore, a simple and rapid indicator is needed to predict the possibility of ICU readmission.

Currently, the predictive indicators for ICU readmission include the acute physiology and chronic health evaluation (APACHE) II score, acute physiology scores (APS), the sequential organ failure assessment (SOFA) score, the stability and workload index for transfer (SWIFT) score, modified early warning score (MEWS), national early warning score (NEWS), and classification algorithms, etc. A retrospective study found that the APACHE II score at discharge was an independent predictor of ICU readmission for surgical intensive care unit patients, particularly early ICU readmission.^[9]^ The APS >40 at discharge was an independent predictor of readmission to the ICU.^[10]^ Another study found that SOFA scores were predictive of being readmitted to the ICU.^[11]^ Kareliusso et al found that SWIFT scores were significantly higher in ICU patients who were readmitted than in ICU patients who were not readmitted and that SWIFT scores ≥ 15 were associated with significantly higher rates of readmission.^[12]^ In a prospective, multicenter study, it was found that both NEWS and MEWS scores were risk factors for ICU readmission incidence and time, furthermore, NEWS was found to be more accurate than MEWS in predicting the prognosis.^[13]^ The classification algorithm of ICU patients’ admission information could predict the risk of readmission and was better than that after ICU discharge.^[14]^ Note that there were no indicators that had high sensitivity and specificity for forecasting poor outcomes.^[15]^ A useful index for predicting ICU readmission is needed to effectively allocate medical resources and reduce the cost of care for hospitals and patients.

Our previous study has found that the Charlson comorbidity index (CCI) was an important predictor of ICU admission in patients with unruptured TAA.^[15]^ There was no research report on CCI and ICU readmission in AA patients currently, so this study further investigated the relationship between CCI and ICU readmission in AA patients and its clinical value.

## 2. Methods

### 2.1. Data source

The data collected in this study came from the Medical Information Mart for Intensive Care- IV (MIMIC-IV) database, which collected the data of more than 190,000 patients and 450,000 inpatients admitted to Beth Israel Deaconess Medical Center between 2008 and 2019.

### 2.2. Inclusion and exclusion criteria

The inclusion criteria were that patients met the following conditions: they were at least 18 years old, had been in hospital for more than 2 days, had been in ICU, had been diagnosed as AAA or TAA, and had a record of CCI. The patients who were diagnosed with ruptured AA, died in the hospital were excluded.

### 2.3. Main observational indicators

The study’s primary outcome indicator was readmission to the ICU for AA patients. The main independent variable was CCI, and the other indicators included length of stay in ICU, demographic factors and indicators measured within 24 hours of admission to the ICU mainly included blood indicators, blood pressure indicators, heart rate, respiratory rate and SOFA. Demographic factors included age, sex, marital status, body mass index (BMI), alcohol, AA category, surgery, hypertension, hyperlipidemia, coronary artery disease, and chronic obstructive pulmonary disease. Marital status was classified as unmarried, married, divorced, and widowed, and BMI was classified as underweight (BMI < 18.5), healthy weight (18.5 ≤ BMI < 25), overweight (25 ≤ BMI < 30), and obese (BMI ≥ 30) according to the World Health Organization’s BMI thresholds. Blood indicators included red blood cell distribution width (RDW), alanine aminotransferase (ALT), aspartate aminotransferase (AST), blood urea nitrogen, creatinine (Cr), anion gap, lactic acid, blood glucose. Blood pressure indicators included systolic blood pressure, diastolic blood pressure, mean arterial pressure, and mean blood pressure.

### 2.4. Statistical analysis

R studio was used for data cleaning and analysis. The data of non-normal distribution were represented by median (P25, P75) and tested by the Mann–Whitney *U* test. The counting data were expressed by frequency and tested by the chi-square test. The receiver operating characteristic (ROC) curve was used to analyze the predictive ability of indicators for ICU readmission of AA patients. Decision Curve Analysis (DCA) was performed to analyze the obtained clinical net benefit of indicators for predicting ICU readmission. The restricted cubic spline (RCS) was employed to analyze the correlation between CCI and ICU readmission of AA patients. Generalized linear regression analysis was further conducted and 3 regression models were established to explore their association by adjusting different variables. The relationship between CCI and ICU readmission of AA patients was also explored using trend analysis by setting CCI as a categorical variable according to its quartile. Their association was evaluated among different subgroups stratifying with surgery requirement (with or without) and AA type (AAA or TAA). After subgroup analysis, the key subgroup can be identified. Then ROC and DCA were performed to analyze the clinical value of CCI for predicting ICU readmission. Finally, the importance order of 18 CCI components on ICU readmission was analyzed using XGBoost regression and random forest regression, respectively. The predictive value of key components on ICU readmission was also analyzed by ROC. *P* < .05 was considered as a significant difference.

## 3. Results

### 3.1. Baseline information on patients

A total of 523 patients with AA were enrolled in the study. Tables 1 and 2 show the baseline information of the patients. Patients with AA who were readmitted to the ICU had higher width of RDW (*P* < .01) and higher CCI (*P* < .001) compared to patients who were admitted to the ICU only once. There were no differences in other indicators between the 2 groups.

**Table 1:** Differences in baseline quantitative information between patients admitted to the ICU once and patients readmitted to the ICU.

Variable	ICU admission once	ICU readmission	*P*
Length of stay in ICU (d)	2.062 (1.263, 3.311)	2.131 (1.268, 4.138)	.447
Age (yr)	71.000 (63.000, 79.000)	73.000 (67.000, 79.000)	.170
SBP (mm Hg)	118.000 (103.000, 131.000)	121.000 (107.000, 137.000)	.082
DBP (mm Hg)	59.000 (52.000, 66.000)	60.000 (50.000, 66.000)	.835
MAP (mm Hg)	79.000 (70.667, 87.333)	81.333 (75.000, 87.667)	.363
MBP (mm Hg)	80.000 (70.000, 89.000)	79.000 (66.000, 87.000)	.733
HR	120.000 (110.000, 130.000)	120.000 (120.000, 130.000)	.224
RR	16.000 (14.000, 20.000)	17.000 (14.000, 20.000)	.519
RDW (%)	13.800 (13.000, 14.700)	14.100 (13.400, 16.100)	.009
ALT (U/L)	19.000 (13.000, 32.000)	19.000 (11.000, 35.000)	.930
AST (U/L)	24.000 (18.000, 41.000)	34.000 (19.000, 56.000)	.167
BUN (mmol/L)	17.000 (13.000, 23.000)	18.000 (13.000, 36.000)	.275
Cr (mg/dL)	0.900 (0.800, 1.200)	0.900 (0.700, 1.600)	.892
AG (mmol/L)	13.000 (11.000, 15.000)	13.000 (12.000, 17.000)	.340
LA (mmol/L)	1.400 (1.100, 2.000)	1.400 (1.100, 2.100)	.718
BG (mg/dl)	109.000 (96.000, 133.000)	99.000 (96.000, 111.000)	.105
SOFA at 24 h	4.000 (2.000, 6.000)	4.000 (2.000, 8.000)	.825
CCI	6.000 (5.000, 8.000)	8.000 (6.000, 9.000)	<.001

AG = anion gap, ALT = alanine aminotransferase, AST = aspartate aminotransferase, BG = blood glucose, BUN = blood urea nitrogen, CCI = Charlson comorbidity index, Cr = creatinine, DBP = diastolic blood pressure, HR = heart rate, LA = lactic acid, MAP = mean arterial pressure, MBP = mean blood pressure, RDE = red blood cell distribution width, RR = respiratory rate, SBP = systolic blood pressure, SOFA = sequential organ failure assessment.

**Table 2:** Differences in baseline qualitative information between patients admitted to the ICU once and patients readmitted to the ICU.

Variable		ICU admission once	ICU readmission	*P*
Surgery (%)	Yes	412 (88.602)	55 (94.828)	.148
	No	53 (11.398)	3 (5.172)	
Sex (%)	Male	316 (67.957)	36 (62.069)	.367
	Female	149 (32.043)	22 (37.931)	
AA types (%)	AAA	253 (54.409)	34 (58.621)	.543
	TAA	212 (45.591)	24 (41.379)	
BMI (%)	Underweight	20 (5.571)	5 (11.628)	.061
	Healthy Weight	79 (22.006)	15 (34.884)	
	Overweight	93 (25.905)	10 (23.256)	
	Obese	167 (46.518)	13 (30.233)	
Marital status (%)	Unmarried	92 (21.346)	15 (27.778)	.250
	Married	240 (55.684)	23 (42.593)	
	Divorced	35 (8.121)	4 (7.407)	
	Widowed	64 (14.849)	12 (22.222)	
Drink (%)	Yes	24 (5.161)	1 (1.724)	.247
	No	441 (94.839)	57 (98.276)	
Hypertension (%)	Yes	257 (55.269)	29 (50.000)	0.447
	No	208 (44.731)	29 (50.000)	
Hyperlipidemia (%)	Yes	179 (38.495)	20 (34.483)	0.553
	No	286 (61.505)	38 (65.517)	
CAD (%)	Yes	270 (58.065)	26 (44.828)	0.055
	No	195 (41.935)	32 (55.172)	
COPD (%)	Yes	105 (22.581)	10 (17.241)	0.355
	No	360 (77.419)	48 (82.759)	

Abbreviations: AA = aortic aneurysm, AAA = abdominal aortic aneurysm, CAD = coronary artery disease, COPD = chronic obstructive pulmonary disease, TAA = thoracic aortic aneurysm.

### 3.2. Clinical value of Charlson comorbidity index in predicting intensive care unit readmission and their association exploration

**Figure 1.**
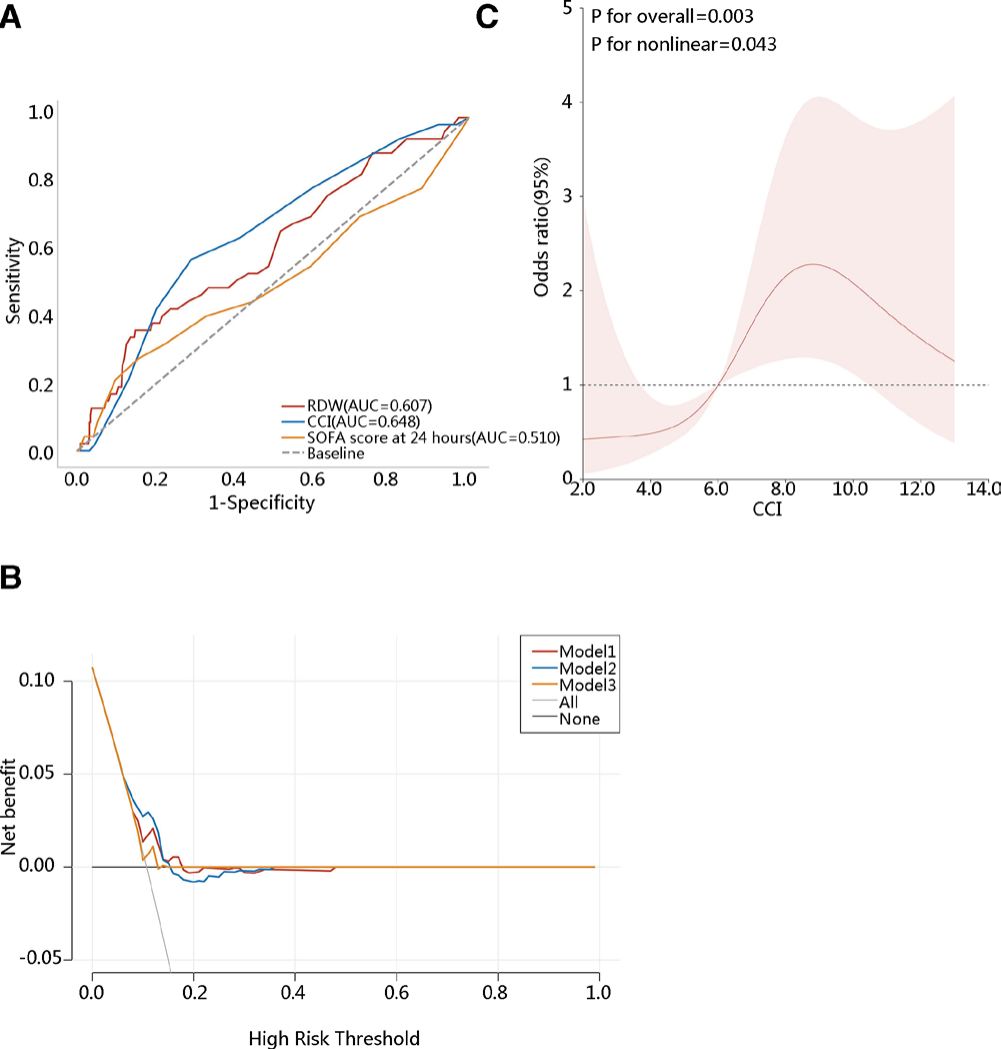
Clinical value of CCI in predicting intensive care unit (ICU) readmission and their association exploration (A) the receiver operating characteristic (ROC) curve, (B) the decision curve analysis (DCA) curve, (C) the RCS analysis. RDE = red blood cell distribution width, CCI = Charlson comorbidity index, SOFA = sequential organ failure assessment,. Model 1: RDW, Model 2: CCI, Model 3: SOFA score at 24 hours.

The importance of RDW and CCI on the ICU readmission was initially found. Then we conducted the ROC analysis to explore their predictive value. As the SOFA score at 24 hours is a commonly used prognostic indicator in clinical practice for adult ICU patients, hence, we also included the SOFA score as a reference variable to better evaluate the prediction performance of RDW and CCI. The area under curve (AUC) for RDW and CCI was 0.607 and 0.648, respectively, while for SOFA score at 24 hours, it was 0.510 (Fig. 1A). The cutoff of CCI was 7.5 (data not shown). The predictive value of RDW and CCI were superior to SOFA, and CCI had the most favorable prediction performance (data not shown). DCA curve showed that when the threshold was about 0.06 to 0.15, the clinical benefit of the CCI for predicting the ICU readmission of AA patients was higher than that of RDW, SOFA score at 24 hours, treat-all and treat none model (Fig. 1B).

Due to the promising value of CCI on ICU readmission, the correlation between them was next explored. The RCS analysis indicated a nonlinear correlation between CCI and ICU readmission (*P* for nonlinear = .043) (Fig. 1C). Subsequently, we investigated this relationship using generalized linear regression and found that CCI was significantly associated with ICU readmission in the crude model (*P* = .003). Their association was still observed in model 2 (*P* = .007) and model 3 (*P* < .001) after adjusting for different variables (Table 3). Our results suggested an independent association between CCI and ICU readmission.

**Table 3:** Generalized linear regression analysis of the relationship between CCI and ICU readmission.

Variable		β (95% CI)	*P*
Model 1	Intercept	0.030 (−0.073, 0.078)	.947
	CCI	0.017 (0.006, 0.027)	.003
Model 2	Intercept	0.021 (−0.136, 0.178)	.793
	CCI	0.017 (0.005–0.030)	.007
	Age	0.000 (−0.003, 0.002)	.745
	Female	0.020 (−0.037, 0.078)	.493
Model 3	Intercept	0.023 (−0.071, 0.116)	.638
	CCI	0.022 (0.010, 0.034)	<.001
	Hypertension	0.007 (−0.050, 0.063)	.818
	Hyperlipidemia	−0.061 (−0.116, −0.006)	.029
	CAD	−0.026 (−0.083, 0.030)	.359
	COPD	−0.067 (−0.134, −0.001)	.049

CAD = coronary artery disease, CCI = Charlson comorbidity index, COPD = chronic obstructive pulmonary disease.

All patients were then divided into 4 groups according to CCI quartiles from smallest to largest, and the relationship between CCI and ICU readmission was also analyzed using the trend test after setting CCI as a categorical variable. As can be seen from Table 4, the risk of ICU readmission increased with higher CCI scores in model 1, model 2, and model 3 (all *P* < .05). The trend analysis suggested their positive association.

**Table 4:** Trend analysis of the relationship between CCI and ICU readmission.

Quartile of CCI	Model 1	Model 2	Model 3
1	1.00 [reference]	1.00 [reference]	1.00 [reference]
2	0.027 [−0.048,0.102]	0.026 [−0.055,0.106]	0.043 [−0.033,0.119]
3	0.057 [−0.015,0.128]	0.058 [−0.022,0.137]	0.089 [0.014,0.163]*
4	0.135 [0.063,0.207]**	0.136 [0.057,0.216]*	0.176 [0.097,0.256]**
*P* for trend	<.001	.001	<.001

Model 2: adjusted age, sex; Model 3: adjusted hypertension, hyperlipidemia, coronary artery disease, chronic obstructive pulmonary disease.

* *P* < .05.

** *P* < .001.

The AA types and surgery requirements were the important factors associated with the clinical outcome of patients. Therefore, we also explored the association of CCI and ICU readmission among different subgroups by stratifying patients with these 2 clinical traits. The subgroup analysis (Table 5) showed that the CCI of patients who underwent surgery was significantly associated with ICU readmission in model 1, model 2, and model 3 (all *P* < .05). Similarly, the CCI of patients with TAA was significantly associated with ICU readmission in model 1 and model 3, while the CCI of patients with AAA was significantly associated with ICU readmission in model 2 and model 3. It followed that the association between CCI and ICU readmission was unanimously observed in AA patients undergoing surgery. However, their association with different AA types was different in different adjusted models.

**Table 5:** The hierarchical analysis of the relationship between CCI and ICU readmission.

Variable			β (95%CI)	*P*
Model 1			0.017 (0.006,0.027)	.003
	AA types	AAA	0.014 (−0.002,0.030)	.090
		TAA	0.022 (0.006,0.038)	.009
	Surgery	With	0.020 (0.008,0.032)	.001
		Without	0.005 (−0.016,0.026)	.643
Model 2			0.017 (0.005,0.030)	.006
	AA types	AAA	0.017 (0.001,0.034)	.042
		TAA	0.013 (−0.007,0.034)	.199
	Surgery	With	0.021 (0.007,0.035)	.003
		Without	0.004 (−0.019,0.026)	.754
Model 3			0.022 (0.010,0.034)	<.001
	AA types	AAA	0.017 (0.001,0.034)	.043
		TAA	0.022 (0.004,0.041)	.019
	Surgery	With	0.027 (0.014,0.040)	<.001
		Without	0.001 (−0.022,0.024)	.921

Abbreviations: AA = aortic aneurysm, AAA = abdominal aortic aneurysm, TAA = thoracic aortic aneurysm.

Model 2: adjusted age, sex; Model 3: adjusted hypertension, hyperlipidemia, coronary artery disease, chronic obstructive pulmonary disease.

### 3.3. Clinical value of Charlson comorbidity index on intensive care unit readmission in thoracic aortic aneurysm who underwent surgery and abdominal aortic aneurysm who underwent surgery

**Figure 2.**
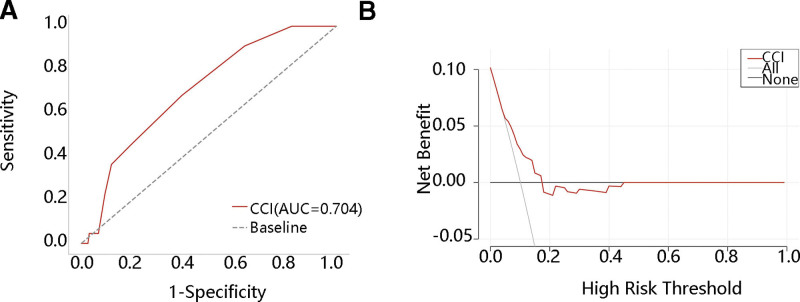
Clinical value of CCI in thoracic aortic aneurysm who underwent surgery (A) the ROC curve, (B) the DCA curve.

We then explored the predictive value of CCI on ICU readmission only in patients undergoing surgery for AAA or TAA. ROC analysis showed that the AUC was 0.704 in TAA patients, with a sensitivity of 0.682 and specificity of 0.605 (Fig. 2A). Figure 2B shows that when the threshold was about 0.05 to 0.18, CCI achieved a greater clinical benefit for predicting ICU readmission in TAA patients who underwent surgery than treat-all and treat none model. The AUC in AAA patients was 0.620, which was lower than that in TAA (data not shown). Therefore, the patients with TAA undergoing surgery were selected for further analysis.

**Figure 3.**
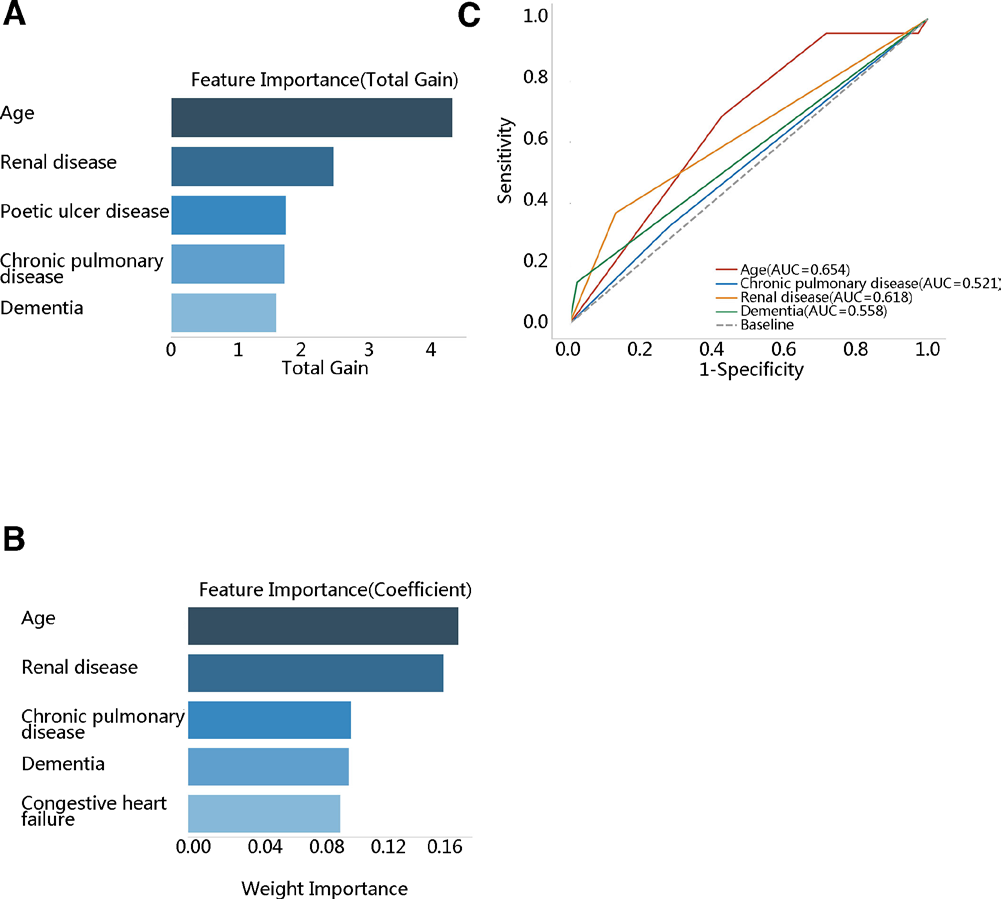
The importance ranking of and the ROC curve for the CCI component in predicting ICU readmission (A) the XGBoost regression, (B) the random forest regression, (C) the ROC curve.

Due to the importance of CCI on the ICU readmission in TAA patients undergoing surgery, we further identified the key CCI components associated with ICU readmission by ranking the importance of CCI components. The top 5 CCI components from XGBoost and random forest regression algorithms were presented in Figure 3A and B, respectively. The XGBoost regression showed that the top 5 feature importance were age, renal disease, poetic ulcer disease, chronic pulmonary disease, and dementia. The random forest regression showed that the top 5 feature importance were age, renal disease, chronic pulmonary disease, dementia, and congestive heart failure. Age, renal disease, chronic lung disease, and dementia were the common components among the 2 algorithms. Then, we evaluated the predictive value of these 4 components on ICU readmission, finding that the AUC of age, renal disease, chronic pulmonary disease, and dementia were 0.654, 0.521, 0.618, and 0.558, respectively (Fig. 3C). Age had more favorable prediction performance than renal disease, chronic pulmonary disease, and dementia.

## 4. Discussion

In this study, we found that CCI and RDW were independent predictors of ICU readmission of AA patients. In addition, CCI had more favorable performance for predicting ICU readmission, especially in TAA patients who underwent surgery.

The RDW is mainly used to predict clinical morbidity and mortality.^[16]^ High RDW was found to be a risk factor for 28-day mortality in critically ill patients over 90 years of age.^[17]^ Jia et al extracted patients with acute kidney injury who were admitted to the ICU for the first time from the MIMIC-III database, after analysis, they found that the higher RDW, the shorter survival time, and the higher mortality rate, RDW was an independent risk factor for patients, and the long-term prognosis of RDW was more effective.^[18]^ Research had shown that there was a linear relationship between the risk of death from acute aortic dissection and the increase in RDW. Specifically, the risk of death increases by 5% for every standard deviation increase in RDW.^[19]^ Less research has been done on its use to predict readmission to the ICU. In this study, we found the relationship that RDW was 1 of the independent predictors of ICU readmission of AA patients. A secondary analysis of a prospective study also found that elevated RDW at ICU discharge was an independent risk factor for ICU readmission after multivariable adjustment.^[20]^ The mechanism behind the predictive ability of RDW for ICU readmission was not entirely clear. This may be linked to the oxidative stress and inflammatory response of patients.^[21]^ We speculated that RDW was widened due to inflammation and oxidative stress, which in turn led to the deterioration of patients’ performance and condition, reduced the ability of ICU patients to deal with complications after discharge,^[20]^ and increased the readmission rate of ICU patients.

CCI is a reliable, high-quality, very sensitive, and effective index measurement standard according to current clinical practice.^[22]^ At present, CCI has been proven to predict the morbidity, mortality, and ICU admission risk of different clinical populations. Soh et al systematically reviewed the morbidity indicators for inpatient mortality prediction and found that high CCI scores predicted a higher risk of death after discharge in patients who were admitted to hospital wards.^[23]^ A study of cardiovascular disease in Australian women found an increased risk of repeat hospitalization and death in women with higher CCI on admission.^[24]^ Liu et al explored the influence of non-cancer factors on the incidence and mortality of glioblastoma multiforme and found that the higher the CCI, the higher the incidence in the total postoperative complications and nervous system.^[25]^ It was found that CCI > 3 is an independent risk factor for accidental ICU admission in patients after radical cystectomy.^[26]^ A few studies focused on the relationship between ICU readmission and CCI. Ranney et al collected the data of trauma patients admitted to the ICU in the past 10 years, analyzed the patients’ age, CCI, and injury severity score, and found that age, CCI, and Injury Severity Score were independently related to ICU readmission.^[27]^ Our research found for the first time that CCI was 1 of the independent predictors of ICU readmission of AA patients. Besides, CCI was significantly associated with ICU readmission in patients with TAA who underwent surgery. We also ranked the importance of CCI components in predicting ICU readmission and found that age was the top 1. TAA is most common in people over the age of 65.^[28]^ To some extent, it also showed that CCI was more accurate in predicting ICU readmission. In addition, the AUC of CCI was greater than the SOFA score at 24 hours. In summary, CCI is a good predictor of ICU readmission. It is recommended that the CCI should be included in the subsequent prediction of the risk of ICU readmission.

Readmission has become a common dimension of medical quality evaluation.^[29]^ Various evaluation indicators and tools have been used to evaluate the risk of readmission in the ICU, mainly at the first discharge. CCI and RDW, which are the indicators for the first stay in the ICU, could predict the risk of ICU readmission, which is more conducive for medical staff to evaluate the patient’s situation and make more effective decisions.

The study has some limitations. Firstly, it did not distinguish whether the ICU readmitted patients were unplanned. Secondly, we had not calculated the readmission interval. However, it is noteworthy that this study is the first to find a significant relationship between CCI and ICU readmission of TAA patients who underwent surgery, which can serve as a reference for future research.

## 5. Conclusions

This study revealed for the first time that RDW and CCI were independently related to the ICU readmission of AA patients. CCI had more favorable prediction performance on ICU readmission than RDW and achieved a better clinical net benefit. CCI showed significant positive association with ICU readmission, especially in TAA patients undergoing surgery. Age was the most important CCI component that correlated with ICU readmission of TAA patients.

## Author contributions

**Conceptualization:** Yu-Fei Zhan.

**Data curation:** Yu-Fei Zhan, Feng Li.

**Formal analysis:** Yu-Fei Zhan, Long-Chuan Wu, Lin Chen.

**Investigation:** Ming-Shuai Han, Guo-Fang Ma.

**Methodology:** Can-Yan Zhu, Yong-Hong Zhong.

**Supervision:** Yong-Hong Zhong.

**Writing – original draft:** Yu-Fei Zhan, Feng Li, Long-Chuan Wu, Lin Chen, Can-Yan Zhu, Ming-Shuai Han, Guo-Fang Ma, Yong-Hong Zhong.

**Writing – review & editing:** Yong-Hong Zhong.
